# The Clinical Course of Patients with Preschool Manifestation of Type 1 Diabetes Is Independent of the HLA DR-DQ Genotype

**DOI:** 10.3390/genes8050146

**Published:** 2017-05-19

**Authors:** Christina Reinauer, Joachim Rosenbauer, Christina Bächle, Christian Herder, Michael Roden, Sian Ellard, Elisa De Franco, Beate Karges, Reinhard W. Holl, Jürgen Enczmann, Thomas Meissner

**Affiliations:** 1 Department of General Pediatrics, Neonatology and Pediatric Cardiology, University Children’s Hospital, Heinrich Heine University Düsseldorf, 40225 Düsseldorf, Germany; 2 Institute for Biometrics and Epidemiology, German Diabetes Center, Leibniz Center for Diabetes Research at Heinrich Heine University Düsseldorf, 40225 Düsseldorf, Germany; Joachim.Rosenbauer@ddz.uni-duesseldorf.de (J.R.); Christina.Baechle@ddz.uni-duesseldorf.de (C.B.); 3 German Center for Diabetes Research (DZD), 85764 München-Neuherberg, Germany; christian.herder@ddz.uni-duesseldorf.de (C.H.); Michael.Roden@ddz.uni-duesseldorf.de (M.R.); bkarges@ukaachen.de (B.K.); reinhard.holl@uni-ulm.de (R.W.H.); 4 Institute for Clinical Diabetology, German Diabetes Center, Leibniz Center for Diabetes Research at Heinrich Heine University Düsseldorf, 40225 Düsseldorf, Germany; 5 Department of Endocrinology and Diabetology, Medical Faculty, Heinrich Heine University Düsseldorf, 40225 Düsseldorf, Germany; 6 Institute of Biomedical and Clinical Science, University of Exeter Medical School, Exeter EX2 5DW, UK; sian.ellard@nhs.net (S.E.); E.De-Franco@exeter.ac.uk (E.D.F.); 7 Division of Endocrinology and Diabetes, Medical Faculty, RWTH Aachen University, 52074 Aachen, Germany; 8 Institute of Epidemiology and Medical Biometry, ZIBMT, University of Ulm, 89069 Ulm, Germany; 9 Institute for Transplantation Diagnostics and Cell Therapeutics, Heinrich Heine University Düsseldorf, 40225 Düsseldorf, Germany

**Keywords:** MHC II, C-peptide, autoimmunity, human leukocyte antigen, diabetes mellitus

## Abstract

Introduction: Major histocompatibility complex class II genes are considered major genetic risk factors for autoimmune diabetes. We analysed Human Leukocyte Antigen (HLA) *DR* and *DQ* haplotypes in a cohort with early-onset (age < 5 years), long term type 1 diabetes (T1D) and explored their influence on clinical and laboratory parameters. Methods: Intermediate resolution *HLA-DRB1*, *DQA1* and *DQB1* typing was performed in 233 samples from the German Paediatric Diabetes Biobank and compared with a local control cohort of 19,544 cases. Clinical follow-up data of 195 patients (diabetes duration 14.2 ± 2.9 years) and residual C-peptide levels were compared between three HLA risk groups using multiple linear regression analysis. Results: Genetic variability was low, 44.6% (104/233) of early-onset T1D patients carried the highest-risk genotype HLA-DRB1*03:01-DQA1*05:01-DQB1*02:01/DRB1*04-DQA1*03:01-DQB1*03:02 (HLA-DRB1*04 denoting *04:01/02/04/05*), and 231 of 233 individuals carried at least one of six risk haplotypes. Comparing clinical data between the highest (*n* = 83), moderate (*n* = 106) and low risk (*n* = 6) genotypes, we found no difference in age at diagnosis (mean age 2.8 ± 1.1 vs. 2.8 ± 1.2 vs. 3.2 ± 1.5 years), metabolic control, or frequency of associated autoimmune diseases between HLA risk groups (each *p* > 0.05). Residual C-peptide was detectable in 23.5% and C-peptide levels in the highest-risk group were comparable to levels in moderate to high risk genotypes. Conclusion: In this study, we saw no evidence for a different clinical course of early-onset T1D based on the HLA genotype within the first ten years after manifestation.

## 1. Introduction

Nowadays, approximately 25–30% of type 1 diabetes (T1D) patients are younger than five years at the time of diagnosis and epidemiological studies reported a continuous rise in the incidence, particularly in preschool children [1,2,3,4,5]. Exposure to genetic, immunological, as well as environmental factors play an important role in disease etiology, but the underlying mechanisms are largely unknown [6].

Although T1D is not a classic inherited disease, and about 80% of T1D manifestations occur in persons without a positive family history for T1D, the individual genetic background is an important factor in the autoimmune process. Genome-wide association studies revealed that approximately half of the genetic risk for T1D is conferred by the genomic region harbouring the major histocompatibility complex (MHC) class II genes, primarily Human Leukocyte Antigen (HLA) *DRB1*, *DQA1*, and *DQB1* [7,8,9]. These genes are encoding cell surface glycoproteins, presenting intra- or extracellular peptides, and play a role in orchestrating the T-cell mediated autoimmune destruction of the pancreatic β-cells [10,11]. While MHC class I molecules are present on virtually all nucleated cells, MHC class II molecules are expressed on B-cells, dendritic cells, macrophages and activated T-cells [6,11]. Both protective alleles and alleles conferring susceptibility have been described for the genes in the *DQ* and *DR* region of the HLA complex. The empirical risk for T1D may be estimated based on family history, and susceptible or protective HLA genes to vary between 0.01% for individuals with no positive family history and protective HLA alleles, and 30–70% for individuals with an affected sibling or twin, positive for high-risk HLA genes [6]. HLA risk alleles are not only associated with a higher risk but also with an earlier onset of diabetes [12,13,14].

Several studies in European-descent populations have addressed the frequency of high-risk alleles, including the highest-risk heterozygous genotype HLA-DRB1*03:01-DQA1*05:01-DQB1*02:01/DRB1*04-DQA1*03:01-DQB1*03:02 (HLA-DRB1*04 summarizing the HLA-DRB1*04:01/02/04/05 alleles), in children younger than six years at the time of T1D diagnosis [9,13,14,15,16]. Even without a positive family history, these children have an increased lifetime risk of about 5% to develop autoimmune diabetes [17]. The risk increases further to up to 40% with a positive family history for diabetes. The prevalence of HLA alleles is highly variable, depending on the ethnic background. HLA class I alleles, e.g., A*24, and B*39, also contribute to the diabetes risk [18]. In adulthood, besides HLA genes, other genes gain relevance. Genome-wide association analyses of adult-onset T1D yielded more than 50 non-HLA genes, such as *PTPN22*, *STAT4*, *CTLA4*, *IL2RA*, *INS*, *ERBB3*, *SH2B3* and *CLEC16A* associated with islet autoimmunity [19,20].

The aim of our study was to examine the *HLA-DR* and *DQ* haplotypes in more than 200 clinically well-characterized children with early-onset diabetes (manifestation before the age of five years) from the German Pediatric Diabetes Biobank with regard to the presence of high-risk HLA genotypes. As the impact of HLA genes on clinical parameters in T1D in follow-up is largely unknown [21,22], we focused on the influence of HLA haplotypes on clinical and laboratory parameters. We aimed to analyse whether high-risk HLA alleles lead to a more severe phenotype.

## 2. Materials and Methods

### 2.1. Study Population

A nationwide, population-based cohort study, called the ‘Clinical Course of Type 1 Diabetes in Children, Adolescents, and Young Adults with Disease Onset in Preschool Age’ study, was initiated in 2009 as part of the German Competence Network on Diabetes Mellitus and integrated into the German Center for Diabetes Research (DZD) as of January 2015 [23]. The study included patients with manifestation before the age of five years between 1993 and 1999, and at least 10 years of diabetes duration. All included patients were diagnosed with T1D by their physicians based on clinical and laboratory parameters.

### 2.2. Inclusion Criteria

Participants were invited by telephone interview to provide biosamples and a random group of 261 individuals donated samples (DNA, plasma, and serum) to the German Pediatric Diabetes Biobank at the German Competence Network/German Diabetes Center, Düsseldorf, Germany. Complete HLA-typing was successful in 233 samples, baseline clinical data (sex, age, migration background) were available for 223 patients, and used for description of clinical data, while conjoined clinical datasets with HLA were available for 195 patients and included in the analysis of the HLA impact on clinical data.

The study was conducted following the principles expressed in the Declaration of Helsinki and approved by the Ethics Committee of the Heinrich Heine University, Düsseldorf (internal study numbers 3431 and 5800). The DPV registry (diabetes prospective follow up, Diabetes Patienten Verlaufsdokumentation) has the ethical approval at the University of Ulm plus local data safety approval.

### 2.3. Variable Assessment

Longitudinal anthropometric (weight, height, body mass index (BMI)), clinical (lipid profile, creatinine, urine albuminuria, blood pressure), and diabetes-related data (frequency of self-monitoring of blood glucose (SMBG), total daily insulin dose, daily insulin dose per kg body weight, insulin therapy (conventional therapy (CT), i.e., ≤3 daily injections; multiple daily injections (MDI), i.e., ≥4 daily injections; continuous subcutaneous insulin infusion (CSII), pump therapy), glycated haemoglobin (HbA1c), events of severe hypoglycaemia or hypoglycaemic coma, hypoglycaemia or diabetic ketoacidosis-related hospitalization and diabetes-related autoimmune disease (thyroiditis, coeliac disease) after diabetes onset were retrospectively obtained from the German/Austrian nationwide DPV registry [24] for the period of 1993–2014 by pseudonymous linkage. 16 out of 223 T1D patients had a migration background (Slovakia, Morocco, Sweden, UK, USA, Poland, Kazakhstan, Russia, Greece, Argentina, Pakistan, Afghanistan, Croatia and Romania (each one patient) and Turkey (two patients)). The 195 clinically well-characterized patients had an average observation period of 12.1 ± 4.3 years (median 13 years, interquartile range (IQR) 9; 17 years) and on average 4.8 ± 1.9 visits with their local diabetes center per year (median 4, IQR 3.5; 6 visits). For each patient, all available data for continuous or ordinal variables were averaged (median) and the number of events of hypoglycaemia or diabetic ketoacidosis were summed up stratified by year of diabetes duration during the follow-up time. Presence of long-term diabetic complications and diabetes-associated autoimmune diseases was assumed in patients if the respective condition was documented at least at one visit during the total individual follow-up time. Standard deviation scores of BMI (BMI-SDS) were determined using German reference data (KIGGS, German Health Interview and Examination Survey for Children and Adolescents [25]) by applying the least-mean-squares (LMS) method [26]. To adjust for between-center variation owing to different laboratory methods, HbA1c measurements were standardized to the Diabetes Control and Complication Trial (DCCT) reference range (4.05–6.05%, 20–42 mmol/mol) using the multiple-of-the-mean method as described previously [24].

### 2.4. Control Population

HLA antigen frequencies of a large regional control population recruited from a centre in North Rhine-Westphalia, Germany, were retrospectively derived from 19,544 randomly selected samples from the Bone Marrow Donor Registry Düsseldorf. As self-reported ethnicity was not available for registry donors, those cases with a known migration background could not be excluded from the control population. The ethnic background of the control and patient group can be expected to be comparable. Thus, we decided to include T1D patients with a known migration background in the analysis in order to maintain a comparable ethnic background. Registry donors underwent HLA typing at the Institute for Transplantation Diagnostics and Cell Therapeutics (ITZ) at the University Hospital Düsseldorf as described below.

### 2.5. Human Leukocyte Antigen Typing

Genomic DNA was extracted from blood samples and purified using QIAamp DNA Blood Midi Kit (Qiagen, Hilden, Germany). HLA-typing was performed in an American Society for Histocompatibility and Immunogenetics (AHSI) accredited laboratory at the Institute for Transplantation Diagnostics and Cell Therapeutics (ITZ) at the University Hospital Düsseldorf. High resolution HLA-typing of the donors in the Bone Marrow Donor Registry Düsseldorf was performed by sequencing-based typing (SBT) of each second exon, as described previously [27]. Complete *HLA-DRB1-DQA1-DQB1* intermediate-resolution typing was performed in 233 DNA specimens from the German Pediatric Diabetes Biobank without changes in setup. HLA-typing of the patients’ samples was performed using the LabType SSO (One Lambda, Kanoga Park, CA, USA) with Luminex technology [28], according to the manufacturer’s protocol. In this test, the second (and third in cases of *DQ*) exon is amplified and analysed. The intermediate typing results which did not belong to the Common and Well Defined Alleles (CWD) group were excluded [29]. Allele Combinations were assessed in the IPD-IMGT/HLA database (Version 3.25.0, accessible online at http://www.ebi.ac.uk/ipd/imgt/hla/ [30]). The *HLA-DRB1-DQA1-DQB1* haplotypes were determined by comparison of the phenotype with well-known haplotype frequencies [31]. The two haplotypes with the highest product of the haplotype frequencies were selected from all possible haplotype combinations in each case. Calculations were performed using an in-house developed computer program (Visual Basic.NET). In ambiguous alleles such as DQB1*02:01 and *02:02*, that were indistinguishable in the SBT-typed controls, we derived the allele from the haplotype combination using this approach. HLA-DRB1*14:01 and *14:54* and DQA1*04:01 and *04:02* were indistinguishable by intermediate resolution typing and are therefore listed as ambiguous alleles.

### 2.6. Neonatal Diabetes Genes

Sanger sequencing of the most common neonatal diabetes genes (*ABCC8*, *KCNJ11*, *INS*) was performed for the eight patients who developed diabetes before the age of 12 months, as previously described [32].

### 2.7. C-peptide

C-peptide in biobank serum specimens was analysed using the Mercodia Ultrasensitive enzyme-linked immuno-sorbent assay (ELISA, Mercodia, Uppsala, Sweden, detection limit 2.5 pmol/L) at the Institute of Clinical Diabetology, Düsseldorf, according to the manufacturer’s protocol (intra-assay coefficient of variation (CV) 1.5%, inter-assay CV 2.9%), as described previously [33].

### 2.8. Statistics

For a simple description of the patients and specific analyses (diabetic complications, associated autoimmune disorders) only the aggregated data of the last documented year of diabetes duration were used, while for further analyses the entire longitudinal dataset was used. For descriptive analysis, mean (standard deviation, SD), median (interquartile range, IQR), and percentages for categorical variables were calculated. Event rates of severe hypoglycaemia, hypoglycaemic coma, hyperglycaemia, or ketoacidosis-associated hospitalization were analysed assuming a Poisson distribution. Continuous and categorical variables were compared between groups by the Wilcoxon test or the χ^2^-(expected number of cases > 5 in each group) or Fisher’s exact test. Additionally, odds ratios were estimated for comparisons of haplotype and genotype frequencies. Multivariable mixed linear (continuous outcome), mixed Poisson (rate outcome) or fixed-effects logistic regression (binary outcome with last year of observation) were applied to adjust for covariates in comparison analyses. Within the regression analyses, F-tests (linear and Poisson regression) or χ^2^-tests (logistic regression) were used to test for statistical significance. Results from regression analyses are presented as adjusted means, event rates per 100 patient years, or percentages, including IQR and respective *p*-values. In all analyses, we did not adjust for multiple testing. *p*-values of two-sided tests < 0.05 were considered statistically significant. All statistical analyses were performed with the statistical software package SAS for Windows, version 9.4 (SAS Institute Inc., Cary, NC, USA).

## 3. Results

### 3.1. Patient Characteristics

Mean age at onset of T1D was 2.8 ± 1.2 years (median 2.8, IQR 1.7; 3.8 years) and mean diabetes duration at the last documented year was 14.2 ± 2.9 years (median 15.0, IQR 13.1; 16.1 years). Males represented 55.2% of the population. The mean HbA1c within the patient group was 8.0 ± 1.4% (64 ± 15 mmol/mol) (median 7.9, IQR 7.1; 8.7%; median 83, IQR 54; 72 mmol/mol). The cohort included 16 (7.2%) patients with a migration background. For insulin administration 48.9% of patients used CSII (continuous subcutaneous insulin infusion), 45.7% used multiple daily injections (MDI), and 5.4% used less than three daily injections (CT).

We analysed the most relevant common neonatal diabetes genes (*KCNJ11*, *ABCC8*, *INS*) to exclude monogenic diabetes in infancy-onset diabetes (*n* = 8 patients with manifestation before age 12 months). No pathogenic mutations were identified.

### 3.2. Comparison of Human Leukocyte Antigen Haplotypes between the Study Group and the Control Population

#### 3.2.1. Prevalence of Human Leukocyte Antigen Haplotype Combinations

**Figure 1:**
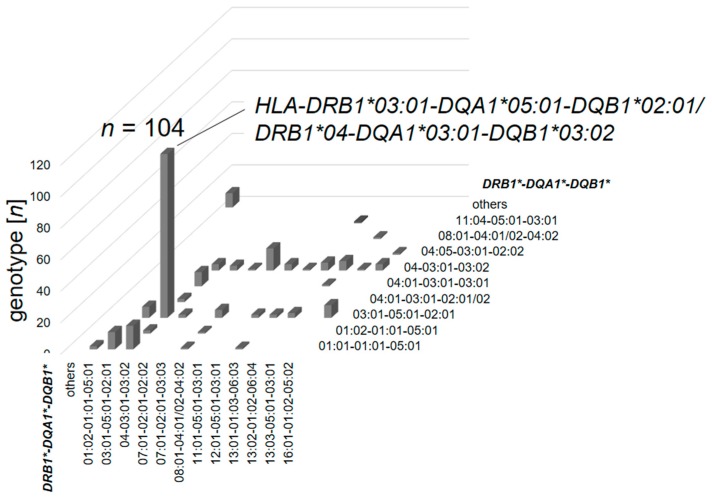
Number of observances of each haplotype combination from the total of the early-onset type 1 diabetes (T1D) cohort (*n* = 233).

The heterozygous haplotype combination HLA-DRB1*03:01-DQA1*05:01-DQB1*02:01/DRB1*04-DQA1*03:01-DQB1*03:02 (DRB1*04 combining the DRB1*04:01/02/04/05 alleles) was the most prevalent genotype and found in 44.6% of the early-onset diabetes subjects (*n* = 104/233, Figure 1). This haplotype combination is named the ‘highest-risk’ genotype. Within the control group the frequency of this haplotype combination was 1.8% (*n* = 343/19,544), resulting in an odds ratio of 45.14 (34.14; 96.99, *p* < 0.001) for the highest-risk genotype in early-onset T1D compared to controls.

#### 3.2.2. Prevalence of HLA Haplotypes

Frequencies of the *DR-DQ* haplotypes were compared between the early-onset T1D cohort and a regional control population (Table 1). Significant susceptibility for T1D was associated with DRB1*03:01-DQA1*05:01-DQB1*02:01 (DR3-DQ2), DRB1*04-DQA1*03:01-DQB1*03:02 (DR4-DQ8), and the rare haplotype DRB1*04:05-DQA1*03:01-DQB1*02:02, as well as DRB1*08:01-DQA1*04:01/02-DQB1*04:02 (DR8-DQ4).

Altogether, 221 out of 233 (94.8%) patients carried at least one of the two main high-risk HLA haplotypes, also abbreviated as DRB1*03 or DRB1*04. While 124 patients exhibited combinations of these, heterozygous (*n* = 104) or homozygous (*n* = 20), 97 patients had one of these two high-risk haplotypes (‘moderate risk’) and only 12 cases (5.2%) did not show either one (‘low risk’).

We separately analysed the second alleles within 97 heterozygous cases showing one high-risk haplotype, DRB1*03 (DR3-DQ2) or DRB1*04 (DR4-DQ8), evaluating the haplotype on the other chromosome (Table 2) and compared with heterozygous controls. Of note, within the DRB1*03/x (DR3-DQ2) heterozygotes, two alleles conveying protection were detected (DRB1*07:01-DQA1*02:01-DQB1*02:02 and DRB1*13:01-DQA1*01:02-DQB1*06:03), while overall the rate of potentially protective alleles was low, even in the second alleles. Genetic heterogeneity was low and most of the second heterozygous alleles were neutral and shared between the two groups. Additional susceptibility was conveyed by DRB1*01:01-DQA1*01:01-DQB1*05:01, DRB1*08:01-DQA1*04:01/02-DQB1*04:02 (see above), and DRB1*13:02-DQA1*01:02-DQB1*06:04 in the DRB1*04/x group, as well as DRB1*16:01-DQA1*01:02-DQB1*05:02 (and less frequently DRB1*09:01-DQA1*03:02-DQB1*03:03) in the DRB1*03/x group.

Investigating the third group of 12 cases, which did not show either DR3-DQ2 or DR4-DQ8, the abovementioned six haplotypes were present in 9 of 12 cases. One patient showed DRB1*04:08-DQA1*03:01-DQB1*03:04, which shares the crucial alanine at position 57 of the DQ beta chain with DQB1*03:02, a known high risk haplotype.

### 3.3. Comparison of Human Leukocyte Antigen Haplotypes between the Study Cohort and Other Adolescent-Onset T1D and T2D Cohorts

**Figure 2:**
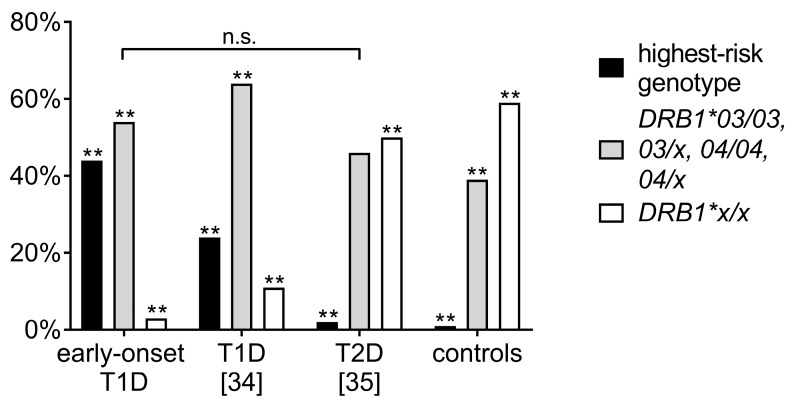
Comparison of the prevalence of Human Leukocyte Antigen (HLA) *DR* risk-groups in the patients with early-onset diabetes before age five years (*n* = 233), and prior analyses of adolescent-onset T1D (*n* = 1445; data from [34]) and type 2 diabetes (T2D) patients (*n* = 109, data from [35]) with a local control population (*n* = 19,544, each ** *p* < 0.001).

HLA haplotype frequencies in early-onset diabetes patients were further compared with previous results from the German/Austrian nationwide diabetes DPV registry, analysing older T1D patients diagnosed before age 20 years (mean age at manifestation 8.3 years; *n* = 1445) and a cohort with juvenile-onset of type 2 diabetes (T2D, mean age at diagnosis 13.8 years; *n* = 109) [34,35]. These cohorts had been in part HLA-typed serologically in the 1970s and 80s [34], while most patients were HLA-typed by Polymerase Chain Reaction (PCR) with sequence-specific primers in the individual laboratories [34,35]. For comparability, the HLA types were subcategorized into three groups. A moderate to high-risk group (DRB1*03/03, or *03/x*, or DRB1*04/04, or *04/x*), and one group with *DR x/x* (the x haplotype denotes other haplotypes, non-03 or -04) were compared to the highest-risk genotype (DRB1*03:01-DQA1*05:01-DQB1*02:01/DRB1*04-DQA1*03:01-DQB1*03:02), as described previously. The prevalence of the highest-risk haplotype was significantly higher in the early-onset T1D cohort compared to both the other aforementioned later onset T1D and T2D patient cohorts. Prevalences in early-onset T1D were significantly different from the other cohorts showing more highest-risk and moderate- to high-risk haplotypes (each *p* < 0.001), while the percentage of patients in the moderate to high risk group did not differ between the early-onset T1D and the T2D group (Figure 2).

### 3.4. Comparison of Clinical Data of the Highest-Risk Haplotype Combination vs. Other Haplotypes

Table 3 shows the comparison of clinical data between patients with the highest-risk genotype (*n* = 86/195) and patients with moderate (*n* = 103) and low risk (*n* = 6) haplotypes. There were no differences in sex distribution (52.3% vs. 54.4% vs. 50% males, *p* = 0.948) or age at manifestation (2.8 ± 1.1 vs. 2.8 ± 1.2 vs. 3.2 ± 1.5 years, *p* = 0.804). The mean age (16.4 ± 3.2 vs. 17.5 ± 3.0 vs. 17.1 ± 2.2 years, *p* = 0.018) and diabetes duration (13.6 ± 3.0 vs. 14.8 ± 3.0 vs. 14.0 ± 2.1 years, *p* = 0.014) at the last year of observation was up to one year shorter in the highest-risk HLA group than in the other groups. Anthropometric parameters, SMBG, total daily insulin use, insulin dosage per kg body weight and blood pressure were comparable between those HLA groups. While HbA1c levels, cholesterol levels, creatinine, and urine albuminuria did not differ between the groups, triglyceride levels were by trend lower in the highest-risk group compared to moderate risk haplotypes (*p* = 0.051). Rates of severe hypoglycaemia, hypoglycaemic coma, and hospitalization related to hypoglycaemia or diabetic ketoacidosis were not significantly different between HLA risk groups. Clinical diagnosis of celiac disease affected 10.6% in the highest-risk, 5.6% in the moderate risk group and 7.0% in the low risk HLA group (Table 3). The prevalence of autoimmune thyroiditis was lower with 8.8 vs. 18.3% in the highest-risk vs. moderate risk group, however not statistically significant. Transglutaminase, thyroid peroxidase (TPO) or thyroglobulin (TG) autoantibody prevalence did not differ significantly between groups.

### 3.5. C-peptide

**Figure 3:**
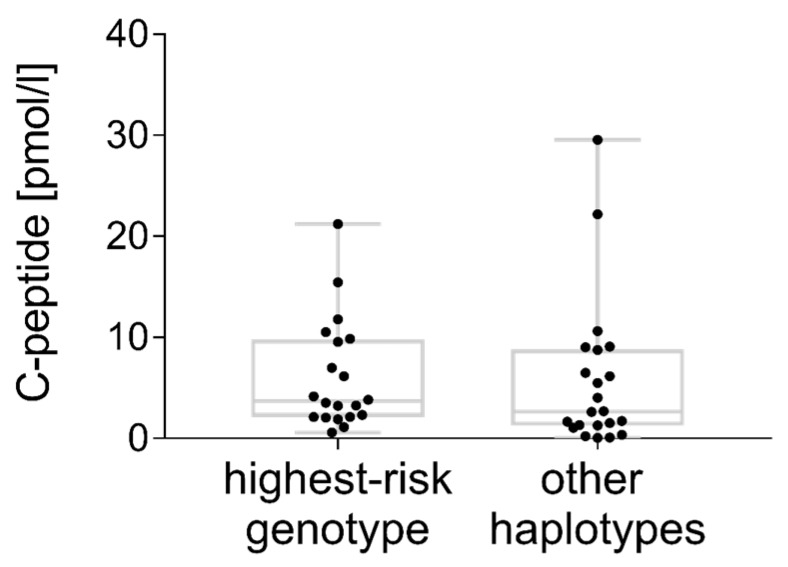
Ultrasensitive residual C-peptide concentrations (pmol/L) compared between patients with the highest-risk haplotype combination and other moderate-risk and low-risk haplotypes, samples taken on average 14.3 ± 2.3 or 13.2 ± 2.6 years after diabetes onset.

C-peptide levels were below the detection limit of standard assays (regularly > 30 pmol/L) and below a clinically relevant threshold (usually defined as > 200 pmol/L). According to the results of the ultrasensitive assay, 25.0% (20/80) of the patients with the highest-risk genotype, 19.8% (19/96) of the patients with a moderate risk genotype and 33.3% (2/6) of the patients with low risk haplotypes had detectable residual C-peptide (*p* = 0.579). C-peptide levels were comparable in all three risk groups (means adjusted for gender and diabetes duration from regression analysis: 6.1 ± 1.5 vs. 6.0 ± 1.6 vs. 5.2 ± 4.9 pmol/L, *p* = 0.982). As there were only two C-peptide levels known in the low risk group, we included them in the ‘other haplotypes’ column (Figure 3).

## 4. Discussion

### 4.1. Aim and Main Findings

We analysed *HLA*-*DRB1-DQA1-DQB1* haplotype frequencies and clinical data with respect to MHC class II risk groups in a German cohort of patients with preschool manifestation of autoimmune diabetes.

### 4.2. Limitations

We acknowledge the limitations of our investigation: T1D was assumed based on the medical history, clinical and laboratory parameters, and was not verified by diabetes-specific autoantibody analyses in all cases. Due to genetic differences between different ethnicities, the results from this European cohort may not be transferred to other populations. Comparability of C-peptide levels between individuals may be limited by random, unstimulated measurement of C-peptide levels.

### 4.3. Highest-Risk Genotype

An earlier clinical onset of T1D is associated with a higher genetic susceptibility [36]. Early-onset T1D was strongly associated with the highest-risk haplotype combination HLA-DRB1*03:01-DQA1*05:01-DQB1*02:01/DRB1*04-DQA1*03:01-DQB1*03:02, affecting 44.6% of the patients in our early-onset cohort. In contrast, the frequency of this haplotype combination in the general Caucasian population is estimated about 2% [37], and was 1.8% in our control population. Our results are in line with previous reports in several European cohorts on manifestation before the age of six years, with 33–52% of individuals sharing this haplotype [7,9,14,15,16,38,39,40]. Noble and Valdes noted that the odds ratios for the diabetes risk were much higher for the heterozygote genotype (OR = 16.6) than for either of the homozygotes (DRB1*03/*03, OR = 6.3; DRB1*04/*04, OR = 5.7) [40]. This was true also in our cohort (DRB1*03/DRB1*04, OR = 45.14 (34.14; 96.99). This synergistic effect favouring the DRB1*03/DRB1*04 genotype was explained by the formation of HLA-DQ8 *trans* (and DQ2 *trans*) dimers in heterozygous individuals [41,42], while this has not yet been demonstrated experimentally.

### 4.4. Other Haplotypes Conferring Risk for T1D

We found an association with DRB1*01:01-DQA1*01:01-DQB1*05:01 (DR1-DQ5), DRB1*16:01-DQA1*01:02-DQB1*05:02 (DR16-DQ5), DRB1*08:01-DQA1*04:01/02-DQB1*04:02 (DR8-DQ4) DRB1*13:02-DQA1*01:02-DQB1*06:04 (plus the rare DRB1*09:01-DQA1*03:02-DQB1*03:03) in the second alleles, showing a predominant role of six haplotypes influencing the risk in 231 of 233 (99.1%) cases. Genetic heterogeneity was low within this early-onset T1D cohort.

For DRB1*08:01-DQA1*04:01/02-DQB1*04:02 a predisposing effect for T1D has been described previously [39], the positive association of DR16-DQ5 in the heterozygotes is a potentially novel finding. Thomson et al. had shown in 1988 that an excess of both DR8 and DR1 occurs in combination with DR4 in individuals lacking the highest-risk heterozygote, while at that time data on the associated DQ allele were not available and DR16-DQ5 was still subsumed under the DR2 serotype [43]. Other authors did not observe a susceptibility for DRB1*01:01-DQA1*01:01-DQB1*05:01 or DRB1*08:01-DQA1*04:01/02-DQB1*04:02 [7,9,14]. As besides HLA II genes the local upregulation of HLA I plays a central role in insulitis and β-cell destruction, MCH class I genotyping data would further our understanding of the underlying genetic risk and should be pursued in future analyses in parallel.

### 4.5. Protection

The absence of DR15-DQ6 in our cohort, which is a common haplotype in the general population (11.9% in the control cohort) clearly shows its protective effect. Strikingly, we also found a lack of protective haplotypes in the secondary alleles and in the ‘low-risk’ group. This well-known phenomenon cannot be easily explained, as mechanisms of protection by HLA class II alleles remain to be determined. Potential mechanisms include tolerance induction to potentially autoantigenic peptides by their stable presentation on cell surfaces, the induction of regulatory T-cells, or deletion of autoreactive T-cells [44,45,46].

### 4.6. Peptide Binding to the MHC Antigen

It is known, that different HLA alleles are associated with a different expression of pancreas-specific autoantibodies. It is likely, but not completely elucidated, that certain MHC II alleles preferentially bind different pancreatic antigenic peptides. Certain amino acid positions in the variable region are particularly important regarding the structure of the binding pocket, determining which potentially pathogenic peptides are presented to the T-cell receptor. Position 57 in the DQ beta-chain (forming the P9-pocket) and residues 13 and 71 in the DR beta-chain (forming the P4 pocket) play the most important role in T1D susceptibility [47]. Alanine at position 57 of the DQ beta-chain confers the highest-risk and is coded by DQB1*02:01/02 and *03:02/04*, while aspartic acid in this position conveys protection [38,46,47,48]. The peptide targets involved in the pathogenesis of T1D are subject of current investigation and include preproinsulin and glutamate decarboxylase (GAD) derived peptides, as well as misfolded or modified proteins [10,48,49,50,51,52]. In celiac disease, also associated with DQB1*02:01 and DQB1*03:02, it is well documented that pathogenic, e.g., gliadin/avenin/glutenin-derived peptides, are recognized by CD4+ T-cells only when bound to HLA DQ2 or DQ8 [53].

### 4.7. The Combination of Alleles Determines the T1D Risk

We would like to point out that it is the combination of *HLA-DRB1*, *DQA1*, and *DQB1* alleles that determines the diabetes risk in our cohort. If we had focused on single alleles, we would have found different risks for T1D for DQB1*02:01 (OR 12.04 (9.97; 14.5), *p* < 0.001) and DRB1*02:02 (no case found in our cohort, OR 0.04 (0.01; 0.15), *p* < 0.001), though DQB1*02:02 and *02:01* have the same amino acid sequence coded by exon 2, coding for the peptide binding motif of the beta-chain of the HLA DQ antigen. Therefore, the associated alpha-chain DQA1*05:01 or the associated HLA-DRB1*03:01, occurring in the linkage disequilibrium with DQB1*02:01, must be responsible for differences in diabetes risk.

Another example shows a predominant role of the DQ antigen instead: DRB1*04:01 occurs linked to DQB1*03:01 or *03:02*. These two *DQ* haplotypes confer a completely different risk for T1D (Table 1), although they both have the same *DRB1* and *DQA1* alleles (considering that both associated DQA1 alleles are identical in exon 2). In this case, the *DRB1* allele is irrelevant for the specificity of peptide presentation, but the *DQB1* plays the pivotal role.

Although both alleles are common in the control population, we did not find the combination DRB1*07:01-DQA1*01:01-DQB1*02:02/DRB1*11:01-DQA1*05:01-DQB1*03:01 within our cohort. This haplotype combination could build a similar DQ complex in *trans* as the high-risk haplotype DRB1*03:01-DQA1*05:01-DQB1*02:01. We can infer that, in this case, it is not the DQ antigen and its peptide binding properties, but DRB1*03:01, that accounts for the susceptibility. We do not see the *DQ2* allele as the crucial risk factor for the development of β-cell autoimmunity.

### 4.8. Clinical Data

One should be aware that we clinically compared patients with the highest-risk haplotype combination to other still high-risk haplotypes, as more than 95% of all patients in the early-onset group had at least one high-risk allele. In contrast to other investigations with broader age ranges analysed [37], we did not observe an age gradient comparing the age groups (0–1, 1–2 years, etc., not shown) nor a difference in age at manifestation between the HLA risk-groups. We examined the associations between *HLA-DRB1-DQA1-DQB1* haplotypes and the clinical characteristics of diabetes in longitudinal data. During a follow-up of an average 12 years of diabetes, we found lower triglyceride levels by trend in the highest-risk group, but cannot derive a clinical implication. In addition to that, we did not detect any clinically-significant differences in clinical, metabolic or laboratory parameters between the HLA risk groups. Previously-reported differences in blood pressure within three years after diagnosis were not confirmed in the present study [22].

The frequency of positive thyroid antibodies in the study cohort was comparable to other studies [54]. Although there is a known association of thyroid autoimmunity with DRB1*03 and DRB1*04 alleles, in our analysis, the clinical diagnosis of autoimmune thyroid disease was less common in T1D patients (8.0% vs. 18.3%) with the highest-risk genotype, missing statistical significance [55]. Our data possibly confirm a minor importance of HLA alleles in the pathogenesis of autoimmune thyroid disease compared to other autoimmune diseases [56].

A previous study reported that patients with positive celiac disease-specific antibodies were significantly younger at diabetes onset [57]. About 90% of celiac disease cases (without comorbid T1D) are associated with DQA1*05:01 and DQB1*02:01 (DQ2) and experience a mechanistically-similar autoimmune process as T1D patients [58]. These alleles may occur in *cis* configuration in association with the DRB1*03 haplotype or in *trans* configuration in a combination with DRB1*07/11/12 positive haplotypes [59], which are rarely seen in T1D.

Within the group of young T1D patients, HLA genotyping is not distinctive for diagnosis of celiac disease, as more than 90% of T1D cases have high-risk *DQ* alleles [58,60,61]. The frequency of celiac disease and associated celiac autoantibody positivity in those with the highest-risk genotype was not statistically significant different from others.

### 4.9. C-peptide

The loss of residual insulin secretion is, next to diabetes duration, related to the age at diabetes onset [62]. After five years of diabetes, 10% of individuals from the SEARCH for Diabetes in Youth study had C-peptide levels above a clinically-relevant threshold (>230 pmol/L) [63]. This indicates that a significant number of β-cells are evading immune attack with implications for future treatment strategies in T1D [64]. Apart from clinically-relevant C-peptide secretion, any detectable C-peptide is also of interest as a marker of surviving functional β-cells. New ultrasensitive C-peptide assays with lower detection limits found residual C-peptides in 10% (serum) to 80% (urine) of T1D individuals, more than 30 years after disease manifestation [30,63]. So far, the influence of the underlying autoimmune process in terms of the genetic background of T1D patients on C-peptide preservation is unknown. With the above-described ultrasensitive assay, random C-peptide was detectable in 23.5% (42/179) of our cases after a mean diabetes duration of 10–20 years. This is lower than that reported previously for patients with onset at a mean of 17.8 years of age, who showed a detectable fasting C-peptide in 38% after 11–20 years of diabetes, suggesting a more severe and complete β-cell destruction in early-onset disease [32]. Within the group of early-onset patients, C-peptide levels did not differ between the HLA risk groups, thus not indicating a more severe phenotype in the subgroup of highest-risk patients.

### 4.10. Clinical Implications

T1D research includes the development of strategies for diagnosing the disease at earlier stages in its natural history with the aim to introduce safe and effective therapeutic options for disease prevention [65]. Large-scale screening studies make use of the predictive value of high-risk HLA DR-DQ haplotypes in newborns (e.g., the ‘Freder1k study in Saxony’ in Germany) to generate large enough groups at risk for primary prevention or interventional studies. Considering that different HLA class II genotypes preferentially bind different autoantigenic peptides, the chances of benefit from a treatment with antigenic peptides (e.g., oral insulin, GAD, Diapep277, etc.) may be, in part, depending on *HLA*-*DR-DQ* genotypes, and this should be addressed in future interventional studies.

## 5. Conclusions

We confirmed the predominant role of the highest-risk haplotype combination for diabetes risk in our early-onset cohort. Genetic heterogeneity was low as far as HLA class II alleles were concerned. Of note, six haplotypes explained part of the diabetes risk in 231 of 233 cases. The importance of protective haplotypes for the prevention of early-onset diabetes is demonstrated by the lack of protective alleles in our cohort, while the mechanism of protection remains elusive. One could hypothesize, that a single protective allele severely reduces the risk to develop T1D in preschool age. A combination of alleles, rather than a single allele or a single amino acid residue in an allele determines the HLA risk for T1D.

HLA status was not associated with significant differences in the clinical course or prevalence of associated autoimmunity in early-onset T1D cases, nor with different levels of residual C-peptide. After more than 10 years of diabetes duration, one in four individuals showed some residual C-peptide, detectable with an ultrasensitive assay. While HLA genes are important to determine the risk to develop early onset T1D, the long-term clinical course in this patient group, independent from the underlying *HLA DR*-*DQ* genotype.

## Figures and Tables

**Table 1:** DRB1-DQA1-DQB1 haplotype frequencies in controls and early-onset type 1 diabetes (T1D) patients and corresponding odds ratios to develop early-onset T1D.

DRB1*	DQA1*	DQB1*	Controls	T1D Patients	Odds Ratio (CI)	*p*
			*n* (%)	*n* (%)		
01:01	01:01	05:01	3582 (9.2)	32 (6.9)	0.73 (0.51; 1.05)	0.087
01:02	01:01	05:01	471 (1.2)	5 (1.1)	0.89 (0.39; 2.08)	0.795
03:01	05:01	02:01	4089 (10.5)	155 (33.3)	4.27 (3.51; 5.17)	<0.001
04:01	03:01	03:02	1801 (4.6)	149 (32.0)	9.73 (7.96; 11.86)	<0.001
04:01	03:01	03:01	989 (2.5)	3 (0.6)	0.25 (0.08; 0.71)	0.010
04:02	03:01	03:02	391 (1.0)	12 (2.6)	2.62 (1.46; 4.63)	<0.001
04:03	03:01	03:02	271 (0.7)	1 (0.2)	0.31 (0.03; 1.69)	0.214
04:04	03:01	03:02	809 (2.1)	19 (4.1)	2.01 (1.28; 3.17)	0.003
04:05	03:01	02:02	36 (0.1)	3 (0.6)	7.03 (2.26; 21.65)	<0.001
04:05	03:01	03:02	111 (0.3)	8 (1.7)	6.13 (2.94; 12.36)	<0.001
07:01	02:01	02:02	3408 (8.7)	5 (1.1)	0.11 (0.05; 0.26)	<0.001
07:01	02:01	03:03	1411 (3.6)	1 (0.2)	0.06 (0.01; 0.31)	<0.001
08:01	04:01/02*	04:02	971 (2.5)	22 (4.7)	1.95 (1.25; 2.97)	0.002
11:01	05:01	03:01	2987 (7.6)	3 (0.6)	0.08 (0.03; 0.22)	<0.001
11:04	05:01	03:01	1363 (3.5)	1 (0.2)	0.06 (0.01; 0.32)	<0.001
12:01	05:01	03:01	757 (1.9)	4 (0.9)	0.44 (0.17; 1.11)	0.092
13:01	01:03	06:03	2737 (7.0)	8 (1.7)	0.23 (0.11; 0.45)	<0.001
13:02	01:02	06:04	1441 (3.7)	12 (2.6)	0.69 (0.39; 1.20)	0.205
13:03	05:01	03:01	542 (1.4)	1 (0.2)	0.15 (0.02; 0.83)	0.031
14:01/54*	01:01	05:03	726 (1.9)	0 (0.0)	0.00 (0; 0.05)	<0.001
15:01	01:02	06:02	4634 (11.9)	0 (0.0)	0.00 (0; 0.39)	0.003
16:01	01:02	05:02	1014 (2.6)	13 (2.8)	1.08 (0.61; 1.86)	0.792
others			4547 (11.6)	9 (1.9)		

**Table 2:** Analysis of the *DRB1-DQA1-DQB1* second alleles in heterozygous HLA DRB1*03/x (DR3-DQ2) and DRB1*04*/x (DR4-DQ8) patients and controls, carrying one high-risk allele. Data were separately analysed for HLA DRB1*03/x (DR3-DQ2) and DRB1*04/x (DR4-DQ8) heterozygous groups.

Patients with DRB1*03/x						
DRB1*	DQA1*	DQB1*	**Controls**	**T1D Patients**	**Odds Ratio (CI)**	***p***
			***n* (%)**	***n* (%)**		
01:01	01:01	05:01	359 (10.5)	11 (29.7)	1.29 (0.62; 2.65)	0.447
07:01	02:01	02:02	332 (9.7)	2 (5.4)	0.19 (0.05; 0.72)	0.009
08:01	04:01/02	04:02	105 (3.1)	5 (13.5)	2.01 (0.83; 4.98)	0.188
09:01	03:02	03:03	23 (0.7)	4 (10.8)	7.55 (2.68; 22.77)	0.007
12:01	05:05	03:01	94 (2.7)	2 (5.4)	0.83 (0.19; 3.18)	0.999
13:01	01:03	06:03	281 (8.2)	2 (5.4)	0.24 (0.06; 0.89)	0.032
13:02	01:02	06:04	142 (4.1)	3 (8.1)	0.81 (0.26; 2.41)	0.999
16:01	01:02	05:02	120 (3.5)	8 (21.6)	3.07 (1.44; 6.69)	0.011
others			1456 (42.5)			
Patients with DRB1*04/x						
DRB1*	DQA1*	DQB1*	**Controls**	**T1D Patients**	**Odds Ratio (CI)**	***p***
			***n* (%)**	***n* (%)**		
01:01	01:01	05:01	170 (11.1)	15 (25.0)	2.68 (1.44; 4.85)	0.003
01:02	01:01	05:01	25 (1.6)	2 (3.3)	2.08 (0,48; 7.88)	0.270
04:01	03:02	03:01	44 (2.9)	2 (3.3)	1.17 (0.27; 4.50)	0.691
07:01	02:01	02:02	124 (8.1)	3 (5.0)	0.59 (0.19; 1.80)	0.624
07:01	02:01	03:03	59 (3.8)	1 (1.7)	0.42 (0.04; 2.38)	0.725
08:01	04:01/02	04:02	67 (4.4)	14 (23.3)	6.66 (3.55; 12.56)	<0.001
08:04	04:01	04:02	3 (0.2)	1 (1.7)	8.65 (0.66; 58.49)	0.142
11:01	05:05	03:01	151 (9.8)	4 (6.7)	0.65 (0.25; 1.77)	0.512
11:03	05:05	03:01	15 (1.0)	1 (1.7)	17.16 (2.98; 84.83)	0.014
12:01	05:05	03:01	26 (1.7)	1 (1.7)	1.97 (0.45; 7.38)	0.291
13:01	01:03	06:03	130 (8.5)	5 (8.3)	0.98 (0.41; 2.38)	0.999
13:02	01:02	06:04	64 (4.2)	6 (10.0)	2.55 (1.13; 5.92)	0.044
13:03	05:05	03:01	18 (1.2)	1 (1.7)	1.43 (0.13; 8.31)	0.520
16:01	01:02	05:02	59 (2.8)	4 (6.7)	1.79 (0.67; 4.78)	0.295
others			397 (25.9%)			

**Table 3:** Adjusted comparisons of clinical data of patients with the highest-risk haplotype combination (HLA-DRB1*03:01-DQA1*05:01-DQB1*02:01/DRB1*04-DQA1*03:01-DQB1*03:02) vs. other HLA-DRB1*03 or DRB1*04 haplotype combinations and those without one of the highest-risk alleles.

	Highest-Risk Haplotype (*n* = 86)		Moderate Risk Haplotype (*n* = 103)		Low Risk Haplotype (*n* = 6)		
	*n* ^2^	% or mean (SE)	*n* ^2^	% or mean (SE)	*n* ^2^	% or mean (SE)	*p* ^1^
Weight (kg)	1008	45.3 (43.5; 47.1)	1286	45.8 (44.2; 47.5)	74	45.5 (38.8; 52.2)	0.917
Height (cm)	1008	146.4 (144.6; 148.2)	1286	146.8 (145.1; 148.4)	74	148.7 (142.0; 155.4)	0.802
BMI-SDS (KIGGS)	1007	0.37 (0.23; 0.52)	1285	0.42 (0.28; 0.55)	74	0.15 (−0.39; 0.70)	0.629
SMBG	925	5.4 (5.2; 5.7)	1228	5.1 (4.9; 5.4)	72	5.3 (4.3; 6.3)	0.304
Insulin dose (IU/d)	1014	38.9 (36.4; 41.4)	1288	40.0 (37.7; 42.3)	75	38.9 (29.5; 48.4)	0.809
Insulin dose per kg bodyweight (IU/kg/d)	1008	0.81 (0.78; 0.85)	1286	0.83 (0.80; 0.86)	74	0.83 (0.70; 0.97)	0.820
HbA1_c_ (%)	1005	7.6 (7.4; 7.8)	1283	7.4 (7.3; 7.6)	73	7.6 (6.9; 8.2)	0.441
HbA1_c_ (mmol/mol)	1005	60 (58; 62)	1283	58 (56; 60)	73	59 (52; 67)	
RR systolic (mmHg)	979	113.0 (111.5; 114.5)	1233	112.7 (111.3; 114.1)	73	113.3 (107.7; 118.8)	0.947
RR diastolic (mmHg)	979	66.9 (65.8; 68.0)	1233	66.8 (65.8; 67.8)	73	64.2 (60.2; 68.3)	0.447
Cholesterol (mg/dL)	748	174.8 (169.9; 179.8)	926	178.2 (173.6; 182.9)	55	174.9 (156.3; 193.4)	0.608
HDL-Cholesterol (mg/dL)	612	62.5 (59.); 65.2)	736	64.2 (61.8; 66.7)	40	61.5 (51.8; 71.2)	0.612
LDL-Cholesterol (mg/dL)	606	96.9 (91.9; 101.9)	732	96.3 (91.7; 101.0)	40	93.8 (75.6; 111.9)	0.942
Triglyceride (mg/dL)	709	98.9 (91.0; 106.9)	874	112.1 (104.8; 119.4)	55	98.4 (69.5; 127.4)	0.051
Creatinine (mg/dL)	718	57.0 (55.3; 58.7)	847	58.5 (57.0; 60.1)	40	55.6 (49.0; 62.3)	0.348
Urine albuminuria (mg/L)	315	11.8 (0.0; 26.0) *	383	16.0 (3.1; 28.9) *	15	1.8 (0.0; 66.0) *	0.852*
Rate of severe hypoglycaemia (1/100 patient years)	1012	18.5 (12.3; 27.9)	1287	28.7 (19.9; 41.4)	75	6.5 (1.2; 35.6)	0.100
Rate of hypoglycaemic coma (1/100 patient years)	1012	3.6 (2.1; 6.1)	1287	5.6 (3.6; 8.7)	75	2.3 (0.3; 19.9)	0.364
Hospitalization rate for hypoglycaemia (1/100 patient years)	1012	1.2 (0.6; 2.4)	1287	1.5 (0.8; 2.7)	75	1.4 (0.1; 15.6)	0.916
Hospitalization rate for ketoacidosis (1/100 patient years)	1012	1.7 (0.9; 3.2)	1287	2.7 (1.6; 4.5)	75	1.4 (0.1; 17.3)	0.494
	*n* ^3^	% or mean (SE)	*n* ^3^	% or mean (SE)	*n* ^3^	% or mean (SE)	*p* ^1^
Microalbuminuria (%) ^4^	80	36.9 (27.0; 48.1)	95	43.0 (33.3; 53.2)	6	17.0 (3.8; 66.2)	0.497
Macroalbuminuria (%) ^4^	80	3.5 (1.1; 10.9)	95	4.9 (1.9; 11.9)	6	7.9 (0.4; 63.7)	0.838
Retinopathy (%) ^4^	82	2.2 (0.5; 9.2)	97	3.8 (1.3; 10.4)	6	8.0 (0.4; 63.4)	0.689
Transglutaminase antibodies (%) ^4^	66	26.3 (16.8; 38.6)	73	20.4 (12.4; 31.7)	4	11.0 (0.5; 76.5)	0.633
Clinical celiac disease (%) ^4^	86	10.6 (5.6; 19.3)	103	4.6 (1.9; 10.7)	6	7.1 (0.4; 62.0)	0.231
TPO antibodies (%) ^4^	72	16.3 (9.3; 27.1)	83	25.0 (16.4; 36.2)	6	5.5 (0.2; 59.0)	0.283
TG antibodies (%) ^4^	56	22.8 (12.8; 37.2)	71	16.9 (9.3; 26.6)	4	4.9 (0.2; 60.2)	0.489
Clinical thyroiditis (%) ^4^	86	8.8 (4.4; 17.0)	103	18.3 (11.7; 27.5)	6	5.8 (0.3; 59.7)	0.148

Data are adjusted estimates from mixed linear, mixed Poisson or fixed effects logistic regression adjusting for sex and diabetes duration. ^1^ F-Test from linear or Poisson regression for continuous or variables and Χ^2^-Test from logistic regression for variables categorical; ^2^ Total number of observation years contributed by patients (varying dependent on performed measurements at clinical visits); ^3^ Total number of patients (varying dependent on performed measurements); ^4^ Only data of the last documented year are used. Diabetic complications and associated autoimmune diseases are defined cumulatively over the individual observation periods per patient. * Not adjusted for sex and diabetes duration. SMBG: frequency of self-monitoring of blood glucose; TPO: thyroid peroxidase; TG: thyroglobulin.

## References

[1] Patterson C.C., Dahlquist G.G., Gyürüs E., Green A., Soltész G., EURODIAB Study Group (2009). Incidence trends for childhood type 1 diabetes in Europe during 1989–2003 and predicted new cases 2005–20: A multicentre prospective registration study. Lancet.

[2] Patterson C.C., Gyürüs E., Rosenbauer J., Cinek O., Neu A., Schober E., Parslow R.C., Joner G., Svensson J., Castell C. (2012). Trends in childhood type 1 diabetes incidence in Europe during 1989–2008: Evidence of non-uniformity over time in rates of increase. Diabetologia.

[3] Vehik K., Hamman R.F., Lezotte D., Norris J.M., Klingensmith G.J., Dabelea D. (2009). Childhood growth and age at diagnosis with Type 1 diabetes in Colorado young people. Diabet. Med..

[4] Knerr I., Wolf J., Reinehr T., Stachow R., Grabert M., Schober E., Rascher W., Holl R.W., DPV Scientific Initiative of Germany and Austria (2005). The ‘accelerator hypothesis’: Relationship between weight, height, body mass index and age at diagnosis in a large cohort of 9248 German and Austrian children with type 1 diabetes mellitus. Diabetologia.

[5] Bendas A., Rothe U., Kiess W., Kapellen T.M., Stange T., Manuwald U., Salzsieder E., Holl R.W., Schoffer O., Stahl-Pehe A. (2015). Trends in Incidence Rates during 1999–2008 and Prevalence in 2008 of Childhood Type 1 Diabetes Mellitus in Germany—Model-Based National Estimates. PLoS ONE.

[6] Morran M.P., Vonberg A., Khadra A., Pietropaolo M. (2015). Immunogenetics of type 1 diabetes mellitus. Mol. Asp. Med..

[7] Erlich H., Valdes A.M., Noble J., Carlson J.A., Varney M., Concannon P., Mychaleckyj J.C., Todd J.A., Bonella P., Fear A.L., Lavant E. (2008). Type Diabetes Genetics Consortium. HLA DR-DQ haplotypes and genotypes and type 1 diabetes risk: Analysis of the type 1 diabetes genetics consortium families. Diabetes.

[8] Noble J.A., Valdes A.M., Cook M., Klitz W., Thomson G., Erlich H.A. (1996). The role of HLA class II genes in insulin-dependent diabetes mellitus: Molecular analysis of 180 Caucasian, multiplex families. Am. J. Hum. Genet..

[9] Lambert A.P., Gillespie K.M., Thomson G., Cordell H.J., Todd J.A., Gale E.A., Bingley P.J. (2004). Absolute risk of childhood-onset type 1 diabetes defined by human leukocyte antigen class II genotype: A population-based study in the United Kingdom. J. Clin. Endocrinol. Metab..

[10] Roep B.O., Peakman M. (2012). Antigen targets of type 1 diabetes autoimmunity. Cold Spring Harb. Perspect. Med..

[11] Bakay M., Pandey R., Hakonarson H. (2013). Genes involved in type 1 diabetes: An update. Genes.

[12] Hathout E.H., Hartwick N., Fagoaga O.R., Colacino A.R., Sharkey J., Racine M., Nelsen-Cannarella S., Mace J.W. (2003). Clinical, autoimmune and HLA characteristics of children diagnosed with type 1 diabetes before 5 years of age. Pediatrics.

[13] Caillat-Zucman S., Garchon H.-J., Timsit J., Assan R., Boitard C., Djilali-Saiah I., Bougnères P., Bach J.F. (1992). Age-dependent HLA genetic heterogeneity of type 1 insulin-dependent diabetes mellitus. J. Clin. Investig..

[14] Emery L.M., Babu S., Bugawan T.L., Norris J.M., Erlich H.A., Eisenbarth G.S., Rewers M. (2005). Newborn HLA-DR, DQ genotype screening: Age- and ethnicity-specific type 1 diabetes risk estimates. Pediatr. Diabet..

[15] Gillespie K.M., Gale E.A.M., Bingley P.J. (2002). High familial risk and genetic susceptibility in early-onset childhood diabetes. Diabetes.

[16] Larsson H.E., Hansson G., Carlsson A., Cederwall E., Jonsson B., Jönsson B., Larsson K., Lynch K., Neiderud J., Lernmark A. (2008). Children developing type 1 diabetes before 6 years of age have increased linear growth independent of HLA genotypes. Diabetologia.

[17] Aly T.A., Ide A., Jahromi M.M., Barker J.M., Fernando M.S., Babu S.R., Yu L., Miao D., Erlich H.A., Fain P.R. (2006). Extreme genetic risk for type 1A diabetes. Proc. Natl. Acad. Sci. USA.

[18] Nejentsev S., Howson J.M., Walker N.M., Szeszko J., Field S.F., Stevens H.E., Reynolds P., Hardy M., King E., Masters J. (2007). Wellcome Trust Case Control Consortium. Localization of type 1 diabetes susceptibility to the MHC class I genes HLA-B and HLA-A. Nature.

[19] Howson J.M., Rosinger S., Smyth D.J., Boehm B.O., Todd J.A., ADBW-END Study Group (2011). Genetic analysis of adult-onset autoimmune diabetes. Diabetes.

[20] Pociot F., Lernmark Å. (2016). Genetic risk factors for type 1 diabetes. Lancet.

[21] Black M.H., Lawrence J.M., Pihoker C., Dolan L.M., Anderson A., Rodriguez B., Marcovina S.M., Mayer-Davis E.J., Imperatore G., Dabelea D. (2013). SEARCH for Diabetes in Youth Study Group. HLA-associated phenotypes in youth with autoimmune diabetes. Pediatr. Diabet..

[22] García Cabezas M.A., Giralt Muiña P., Fernández Valle B., Benito López P. (2010). Outcome differences in pediatric patients with type 1 diabetes mellitus depending on their HLA-DQ genotypes. Med. Clin..

[23] Stahl A., Straßburger K., Lange K., Bächle C., Holl R.W., Giani G., Rosenbauer J. (2012). Health-related quality of life among German youths with early-onset and long-duration type 1 diabetes. Diabet. Care.

[24] Karges B., Rosenbauer J., Kapellen T., Wagner V.M., Schober E., Karges W., Holl R.W. (2014). Hemoglobin A1c Levels and risk of severe hypoglycemia in children and young adults with type 1 diabetes from Germany and Austria: A trend analysis in a cohort of 37,539 patients between 1995 and 2012. PLoS Med..

[25] Rosario A.S., Kurth B.M., Stolzenberg H., Ellert U., Neuhauser H. (2010). Body mass index percentiles for children and adolescents in Germany based on a nationally representative sample (KiGGS 2003–2006). Eur. J. Clin. Nutr..

[26] Cole T.J., Green P.J. (1992). Smoothing reference centile curves: The LMS method and penalized likelihood. Stat. Med..

[27] Knipper A.J., Hakenberg P., Enczmann J., Kuhröber A., Kiesel U., Kögler G., Wernet P. (2000). HLA-DRB1,3,4,5 and -DQB1 allele frequencies and HLA-DR/DQ linkage disequilibrium of 231 German caucasoid patients and their corresponding 821 potential unrelated stem cell transplants. Hum. Immunol..

[28] Dalva K., Beksac M. (2007). HLA typing with sequence-specific oligonucleotide primed PCR (PCR-SSO) and use of the Luminex technology. Methods Mol. Med..

[29] Mack S.J., Cano P., Hollenbach J.A., He J., Hurley C.K., Middleton D., Moraes M.E., Pereira S.E., Kempenich J.H., Reed E.F. (2013). Common and well-documented HLA alleles: 2012 update to the CWD catalogue. Tissue Antigens.

[30] Robinson J., Halliwell J.A., Hayhurst J.D., Flicek P., Parham P., Marsh S.G. (2015). The IPD and IMGT/HLA database: allele variant databases. Nucleic Acids Res..

[31] Klitz W., Maiers M., Spellman S., Baxter-Lowe L.A., Schmeckpeper B., Williams T.M., Fernandez-Viña M. (2003). New HLA haplotype frequency reference standards: High-resolution and large sample typing of HLA DR-DQ haplotypes in a sample of European Americans. Tissue Antigens.

[32] Rubio-Cabezas O., Flanagan S.E., Damhuis A., Hattersley A.T., Ellard S. (2012). K(ATP) channel mutations in infants with permanent diabetes diagnosed after 6 months of life. Pediatr. Diabet..

[33] Wang L., Lovejoy N.F., Faustman D.L. (2012). Persistence of prolonged C-peptide production in type 1 diabetes as measured with an ultrasensitive C-peptide assay. Diabet. Care.

[34] Awa W.L., Boehm B.O., Kapellen T., Rami B., Rupprath P., Marg W., Becker M., Holl R.W., DPV-Wiss Study Group and the German Competence Network Diabetes Mellitus (2010). HLA-DR genotypes influence age at disease onset in children and juveniles with type 1 diabetes mellitus. Eur. J. Endocrinol..

[35] Awa W.L., Boehm B.O., Rosinger S., Achenbach P., Ziegler A.G., Krause S., Meissner T., Wiegand S., Reinehr T., DPV Initiative and the German BMBF Competence Networks Diabetes Mellitus and Obesity (2013). HLA-typing, clinical, and immunological characterization of youth with type 2 diabetes mellitus phenotype from the German/Austrian DPV database. Pediatr. Diabet..

[36] Komulainen J., Kulmala P., Savola K., Lounamaa R., Ilonen J., Reijonen H., Knip M., Akerblom H.K. (1999). Clinical, autoimmune, and genetic characteristics of very young children with type 1 diabetes. Childhood Diabetes in Finland (DiMe) Study Group. Diabet. Care.

[37] Rewers M., Bugawan T.L., Norris J.M., Blair A., Beaty B., Hoffman M., McDuffie R.S., Hamman R.F., Klingensmith G., Eisenbarth G.S. (1996). Newborn screening for HLA markers associated with IDDM: Diabetes autoimmunity study in the young (DAISY). Diabetologia.

[38] Roark C.L., Anderson K.M., Simon L.J., Schuyler R.P., Aubrey M.T., Freed B.M. (2014). Multiple HLA epitopes contribute to type 1 diabetes susceptibility. Diabetes.

[39] Thomson G., Valdes A.M., Noble J.A., Kockum I., Grote M.N., Najman J., Erlich H.A., Cucca F., Pugliese A., Steenkiste A. (2007). Relative predispositional effects of HLA class II DRB1-DQB1 haplotypes and genotypes on type 1 diabetes: A meta-analysis. Tissue Antigens.

[40] Noble J.A., Valdes A.M. (2011). Genetics of the HLA region in the prediction of type 1 diabetes. Curr. Diabet. Rep..

[41] Koeleman B.P., Lie B.A., Undlien D.E., Dudbridge F., Thorsby E., de Vries R.R., Cucca F., Roep B.O., Giphart M.J., Todd J.A. (2004). Genotype effects and epistasis in type 1 diabetes and HLA-DQ trans dimer associations with disease. Genes Immun..

[42] Van Lummel M., Duinkerken G., van Veelen P.A., de Ru A., Cordfunke R., Zaldumbide A., Gomez-Touriño I., Arif S., Peakman M., Drijfhout J.W. (2014). Posttranslational modification of HLA-DQ binding islet autoantigens in type 1 diabetes. Diabetes.

[43] Thomson G., Robinson W.P., Kuhner M.K., Joe S., MacDonald M.J., Gottschall J.L., Barbosa J., Rich S.S., Bertrams J., Baur M.P. (1988). Genetic heterogeneity, modes of inheritance, and risk estimates for a joint study of Caucasians with insulin-dependent diabetes mellitus. Am. J. Hum. Genet..

[44] Schmidt D., Amrani A., Verdaguer J., Bou S., Santamaria P. (1999). Autoantigen-independent deletion of diabetogenic CD4+ thymocytes by protective MHC class II molecules. J. Immunol..

[45] Miyadera H., Ohashi J., Lernmark Å., Kitamura T., Tokunaga K. (2015). Cell-surface MHC density profiling reveals instability of autoimmunity-associated HLA. J. Clin. Investig..

[46] Pugliese A., Boulware D., Yu L., Babu S., Steck A.K., Becker D., Rodriguez H., DiMeglio L., Evans-Molina C., Harrison L.C. (2016). Type Diabetes TrialNet Study Group. HLA-DRB1*15:01-DQA1*01:02-DQB1*06:02 Haplotype Protects Autoantibody-Positive Relatives from Type 1 Diabetes Throughout the Stages of Disease Progression. Diabetes.

[47] Hu X., Deutsch A.J., Lenz T.L., Onengut-Gumuscu S., Han B., Chen W.M., Howson J.M., Todd J.A., de Bakker P.I., Rich S.S. (2015). Additive and interaction effects at three amino acid positions in HLA-DQ and HLA-DR molecules drive type 1 diabetes risk. Nat. Genet..

[48] Boehm B.O., Manfras B., Rosak C., Schöffling K., Trucco M. (1991). Aspartic acid at position 57 of the HLA-DQ beta chain is protective against future development of insulin-dependent (type 1) diabetes mellitus. Klin. Wochenschr..

[49] Harfouch-Hammoud E., Walk T., Otto H., Jung G., Bach J.F., van Endert P.M., Caillat-Zucman S. (1999). Identification of peptides from autoantigens GAD65 and IA-2 that bind to HLA class II molecules predisposing to or protecting from type 1 diabetes. Diabetes.

[50] Durinovic-Belló I., Schlosser M., Riedl M., Maisel N., Rosinger S., Kalbacher H., Deeg M., Ziegler M., Elliott J., Roep B.O. (2004). Pro- and anti-inflammatory cytokine production by autoimmune T cells against preproinsulin in HLA-DRB1*04, DQ8 Type 1 diabetes. Diabetologia.

[51] Knight R.R., Dolton G., Kronenberg-Versteeg D., Eichmann M., Zhao M., Huang G.C., Beck K., Cole D.K., Sewell A.K., Skowera A. (2015). A distinct immunogenic region of glutamic acid decarboxylase 65 is naturally processed and presented by human islet cells to cytotoxic CD8 T cells. Clin. Exp. Immunol..

[52] Kracht M.J., Zaldumbide A., Roep B.O. (2016). Neoantigens and microenvironment in type 1 diabetes: Lessons from antitumor immunity. Trends Endocrinol. Metab..

[53] Sollid L.M., Qiao S.W., Anderson R.P., Gianfrani C., Koning F. (2012). Nomenclature and listing of celiac disease relevant gluten T-cell epitopes restricted by HLA-DQ molecules. Immunogenetics.

[54] Warncke K., Fröhlich-Reiterer E.E., Thon A., Hofer S.E., Wiemann D., Holl R.W., DPV Initiative of the German Working Group for Pediatric Diabetology, German BMBF Competence Network for Diabetes Mellitus (2010). Polyendocrinopathy in children, adolescents, and young adults with type 1 diabetes: A multicenter analysis of 28,671 patients from the German/Austrian DPV-Wiss database. Diabet. Care.

[55] Zeitlin A.A., Heward J.M., Newby P.R., Carr-Smith J.D., Franklyn J.A., Gough S.C., Simmonds M.J. (2008). Analysis of HLA class II genes in Hashimoto’s thyroiditis reveals differences compared to Graves’ disease. Genes Immun..

[56] Jenkins D., Penny M.A., Fletcher J.A., Jacobs K.H., Mijovic C.H., Franklyn J.A., Sheppard M.C. (1992). HLA class II gene polymorphism contributes little to Hashimoto’s thyroiditis. Clin. Endocrinol..

[57] Fröhlich-Reiterer E.E., Hofer S., Kaspers S., Herbst A., Kordonouri O., Schwarz H.P., Schober E., Grabert M., Holl R.W., DPV-Wiss Study Group (2008). Screening frequency for celiac disease and autoimmune thyroiditis in children and adolescents with type 1 diabetes mellitus—Data from a German/Austrian multicentre survey. Pediatr. Diabet..

[58] Bakker S.F., Tushuizen M.E., von Blomberg B.M., Bontkes H.J., Mulder C.J., Simsek S. (2016). Screening for coeliac disease in adult patients with type 1 diabetes mellitus: Myths, facts and controversy. Diabetol. Metab. Syndr..

[59] Monsuur A.J., Wijmenga C. (2006). Understanding the molecular basis of celiac disease: What genetic studies reveal. Ann. Med..

[60] Elias J., Hoorweg-Nijman J.J., Balemans W.A. (2015). Clinical relevance and cost-effectiveness of HLA genotyping in children with Type 1 diabetes mellitus in screening for coeliac disease in The Netherlands. Diabet. Med..

[61] Gutierrez-Achury J., Romanos J., Bakker S.F., Kumar V., de Haas E.C., Trynka G., Ricaño-Ponce I., Steck A., Chen W.M., Type 1 Diabetes Genetics Consortium (2015). Contrasting the Genetic Background of Type 1 Diabetes and Celiac Disease Autoimmunity. Diabet. Care.

[62] Törn C., Landin-Olsson M., Lernmark A., Palmer J.P., Arnqvist H.J., Blohmé G., Lithner F., Littorin B., Nyström L., Scherstén B. (2000). Prognostic factors for the course of beta cell function in autoimmune diabetes. J. Clin. Endocrinol. Metab..

[63] Greenbaum C.J., Anderson A.M., Dolan L.M., Mayer-Davis E.J., Dabelea D., Imperatore G., Marcovina S., Pihoker C., SEARCH Study Group (2009). Preservation of beta-cell function in autoantibody-positive youth with diabetes. Diabet. Care.

[64] Oram R.A., Jones A.G., Besser R.E., Knight B.A., Shields B.M., Brown R.J., Hattersley A.T., McDonald T.J. (2014). The majority of patients with long-duration type 1 diabetes are insulin microsecretors and have functioning beta cells. Diabetologia.

[65] Ziegler A.G., Bonifacio E., Powers A.C., Todd J.A., Harrison L.C., Atkinson M.A. (2016). Type 1 Diabetes Prevention: A Goal Dependent on Accepting a Diagnosis of an Asymptomatic Disease. Diabetes.

