# Correction: Reinauer et al., The Clinical Course of Patients with Preschool Manifestation of Type 1 Diabetes Is Independent of the HLA DR-DQ Genotype. *Genes* 2017, *8*, 146

**DOI:** 10.3390/genes9010013

**Published:** 2018-01-03

**Authors:** Christina Reinauer, Joachim Rosenbauer, Christina Bächle, Christian Herder, Michael Roden, Sian Ellard, Elisa De Franco, Beate Karges, Reinhard W. Holl, Jürgen Enczmann, Thomas Meissner

**Affiliations:** 1 Department of General Pediatrics, Neonatology and Pediatric Cardiology, University Children’s Hospital, Heinrich Heine University Düsseldorf, 40225 Düsseldorf, Germany; 2 Institute for Biometrics and Epidemiology, German Diabetes Center, Leibniz Center for Diabetes Research at Heinrich Heine University Düsseldorf, 40225 Düsseldorf, Germany; Joachim.Rosenbauer@ddz.uni-duesseldorf.de (J.R.); Christina.Baechle@ddz.uni-duesseldorf.de (C.B.); 3 German Center for Diabetes Research (DZD), 85764 München-Neuherberg, Germany; christian.herder@ddz.uni-duesseldorf.de (C.H.); Michael.Roden@ddz.uni-duesseldorf.de (M.R.); bkarges@ukaachen.de (B.K.); reinhard.holl@uni-ulm.de (R.W.H.); 4 Institute for Clinical Diabetology, German Diabetes Center, Leibniz Center for Diabetes Research at Heinrich Heine University Düsseldorf, 40225 Düsseldorf, Germany; 5 Department of Endocrinology and Diabetology, Medical Faculty, Heinrich Heine University Düsseldorf, 40225 Düsseldorf, Germany; 6 Institute of Biomedical and Clinical Science, University of Exeter Medical School, Exeter EX2 5DW, UK; sian.ellard@nhs.net (S.E.); E.De-Franco@exeter.ac.uk (E.D.F.); 7 Division of Endocrinology and Diabetes, Medical Faculty, RWTH Aachen University, 52074 Aachen, Germany; 8 Institute of Epidemiology and Medical Biometry, ZIBMT, University of Ulm, 89069 Ulm, Germany; 9 Institute for Transplantation Diagnostics and Cell Therapeutics, Heinrich Heine University Düsseldorf, 40225 Düsseldorf, Germany

The article entitled “The Clinical Course of Patients with Preschool Manifestation of Type 1 Diabetes is Independent of the HLA DR-DQ Genotype” contained a calculation error in Table 2 and the statistical methods used were not completely described. The additional information *(in italics)* and corrected Table 2 are given below:

The LMS method on page 3, l. 38 denotes the Lambda-Mu-Sigma method, not the least-mean-squares method. Alongside SAS, we used Graphpad Prism 7 with the Baptista Pike method for the estimation of confidence intervals (CI) for odds ratios.

In Table 1 for DRB1*14:01/54*-DQA1*01:01-DQB1*05:03, the OR is 0.00 (0; *0.39*), *p = 0.003*, while for DRB1*15:01-DQA1*01:02-DQB1*06:02, the OR is 0.00 (0; *0.05*), *p < 0.001*, and these were previously interchanged.

For clarification, we would like to add in brackets on page 6, ll. 7 f.: While 124 patients exhibited combinations of these, heterozygous (*n* = 104) or homozygous (*n* = 20, including two cases with a regular DR4 high-risk haplotype in combination with DRB1*04:05-DQA1*03:01-DQB1*02:02), 97 patients had one of these two high-risk haplotypes (‘moderate risk’) and only 12 cases (5.2%) did not show either one (‘low risk’).

The corrected Table 2 is given below. We accordingly reformatted the paragraph in the results section on page 6, ll. 13 ff.: 

*Of note, potentially protective alleles were missing, even in the second alleles*. Genetic heterogeneity was low and most of the second heterozygous alleles were neutral and shared between the two groups. Additional susceptibility was conveyed by DRB1*01:01-DQA1*01:01-DQB1*05:01 and DRB1*08:01-DQA1*04:01/02-DQB1*04:02 in both groups, as well as DRB1*16:01-DQA1*01:02-DQB1*05:02 and less frequently DRB1*09:01-DQA1*03:02-DQB1*03:03 in the DRB1*03/x group, while DRB1*13:02-DQA1*01:02-DQB1*06:04 just failed significance in the DRB1*04/x group. Investigating the third group of 12 cases, which did not show either DR3-DQ2 or DR4-DQ8, the abovementioned five haplotypes were present in nine of 12 cases.

As significance is missing for the allele DRB1*13:02-DQA1*01:02-DQB1*06:04, it has to be deleted in the discussion section on page 10, l. 29.

## Figures and Tables

**Table 2:** Analysis of the DRB1-DQA1-DQB1 second alleles in heterozygous DRB1*03/x (DR3-DQ2) and HLA DRB1*04/x (DR4-DQ8) patient and controls, carrying one high-risk allele. Data were separately analysed for HLA DRB1*03/x (DR3-DQ2) and DRB1*04/x (DR4/DQ8) heterozygous groups.

Patients with DRB1*03/x						
DRB1*	DQA1*	DQB1*	**Controls**	**T1D Patients**	**Odds Ratio (CI)**	***p***
			***n* (%)**	***n* (%)**		
01:01	01:01	05:01	359 (10.5)	11 (29.7)	3.62 (1.76; 7.39)	<0.001
07:01	02:01	02:02	332 (9.7)	2 (5.4)	0.53 (0.13; 1.98)	0.575
08:01	04:01/02	04:02	105 (3.1)	5 (13.5)	4.95 (2.05; 12.22)	0.006
09:01	03:02	03:03	23 (0.7)	4 (10.8)	17.95 (6.37; 53.99)	<0.001
12:01	05:05	03:01	94 (2.7)	2 (5.4)	2.03 (0.47; 7.78)	0.274
13:01	01:03	06:03	281 (8.2)	2 (5.4)	0.64 (0.15; 2.39)	0.765
13:02	01:02	06:04	142 (4.1)	3 (8.1)	2.04 (0.65; 6.03)	0.200
16:01	01:02	05:02	120 (3.5)	8 (21.6)	7.61 (3.56; 16.52)	<0.001
others			1972 (57.5)			
Patients with DRB1*04/x (DRB1*04 combining the DRB1*04:01/02/04/05 alleles, see text)						
DRB1*	DQA1*	DQB1*	**Controls**	**T1D Patients**	**Odds Ratio (CI)**	***p***
			***n* (%)**	***n* (%)**		
01:01	01:01	05:01	291 (11.6)	15 (25.0)	2.55 (1.39; 4.53)	0.001
01:02	01:01	05:01	36 (1.4)	2 (3.3)	2.37 (0.55; 9.34)	0.221
04:01	03:02	03:01	44 (1.8)	2 (3.3)	1.94 (0.45; 7.44)	0.291
07:01	02:01	02:02	260 (10.3)	3 (5.0)	0.46 (0.15; 1.35)	0.276
07:01	02:01	03:03	100 (4.0)	1 (1.7)	0.41 (0.04; 2.24)	0.730
08:01	04:01/02	04:02	202 (8.0)	14 (23.3)	3.48 (1.91; 6.38)	<0.001
08:04	04:01	04:02	8 (0.3)	1 (1.7)	5.31 (0.47; 35.27)	0.192
11:01	05:05	03:01	254 (10.1)	4 (6.7)	0.64 (0.24; 1.69)	0.515
11:03	05:05	03:01	28 (1.1)	1 (1.7)	1.51 (0.14; 9.12)	0.497
12:01	05:05	03:01	53 (2.1)	1 (1.7)	0.79 (0.08; 4.46)	>0.999
13:01	01:03	06:03	209 (8.3)	5 (8.3)	1.00 (0.43; 2.39)	>0.999
13:02	01:02	06:04	118 (4.7)	6 (10.0)	2.26 (1.03; 5.30)	0.058
13:03	05:05	03:01	40 (1.6)	1 (1.7)	1.05 (0.10; 6.08)	0.623
16:01	01:02	05:02	90 (3.6)	4 (6.7)	1.92 (0.73; 5.00)	0.278
others			781 (31.1)			

